# Regulation of the chemokine receptors CXCR4 and ACKR3 by receptor activity-modifying proteins

**DOI:** 10.1016/j.jbc.2024.108055

**Published:** 2024-12-09

**Authors:** Fabian Pfersdorf, Lucas Romanazzi, Mette Marie Rosenkilde, Martin Gustavsson

**Affiliations:** Department of Biomedical Sciences, University of Copenhagen, Copenhagen, Denmark

**Keywords:** G protein-coupled receptor (GPCR), chemokine receptor, receptor activity-modifying proteins (RAMPs), ACKR3, CXCR4, CXCL12, protein-protein interaction, receptor internalization, arrestin recruitment, signal transduction

## Abstract

The chemokine CXCL12 and its two cognate receptors—CXCR4 and ACKR3—are key players in various homeostatic and pathophysiological processes, including embryonic development, autoimmune diseases, tissue repair, and cancer. Recent reports identified an interaction of CXCR4 and ACKR3 with receptor activity-modifying proteins (RAMPs), and RAMP3 has been shown to facilitate ACKR3’s recycling properties. Yet, the functional effects of RAMPs on the CXCL12 signaling axis remain largely elusive. Here, we characterize the effects of RAMPs on CXCR4 and ACKR3 function. We show that, in the absence of a ligand, RAMPs do not affect the cell membrane localization or constitutive internalization of the two receptors. RAMP3 inhibits ligand-stimulated internalization of ACKR3, which retains the receptor at the membrane and inhibits its ability to scavenge CXCL12. In addition, while cAMP inhibition by CXCR4 is unaffected by RAMPs, basal and ligand-stimulated β-arrestin recruitment to both CXCR4 and ACKR3 is reduced in the presence of RAMP3 due to complex formation at the cell surface. The effects on ACKR3 are observed for chemokine, small molecule, and peptide agonists as well as for a N-terminal truncated receptor variant, suggesting that RAMP regulation involves contacts with the transmembrane domain of the receptor. Taken together, our results show that RAMPs regulate the CXCL12 signaling axis by directly interfering with receptor function. These findings could have direct implications for the interplay between receptors *in vivo* as well as future drug design in the therapeutic targeting of the CXCL12 signaling axis.

The CXC chemokine CXCL12 induces migration and activation of stem cells, endothelial cells, and most leukocytes and, therefore, constitutes a crucial component in homeostatic and inflammatory processes, such as embryogenesis, angiogenesis, and wound healing (1). Due to the critical functions of CXCL12, its dysregulation has further been correlated with a variety of pathological conditions, including viral infections, neurodegenerative diseases, and cancer (2).

CXCL12 exerts its effects by binding and activation of (at least) 2 G protein-coupled receptors (GPCRs), the Atypical Chemokine Receptor 3 (ACKR3, also referred to as CXCR7) and the CXC chemokine receptor 4 (CXCR4). Both receptors are part of the chemokine receptor subgroup of class A GPCRs, and while CXCR4 activation leads to canonical G protein signaling, β-arrestin recruitment, and receptor internalization, ACKR3 recruits arrestin and internalizes, but is unable to activate G proteins (hence the name “*atypical*”) (3, 4). ACKR3’s main function is ascribed to the internalization and consequent degradation of extracellular ligands, including the chemokines CXCL12 and CXCL11, as well as opioid peptides (5), adrenomedullin and adrenomedullin-derived peptides (6, 7). Due to this physiological function, ACKR3 is commonly referred to as a scavenging receptor. Its role in signaling, however, is still debated (4, 8, 9).

Given the quasi-ubiquitous functions of CXCL12, the signaling axis around CXCL12/CXCR4/ACKR3 is involved in a plethora of diseases and developmental processes (1, 2). Yet, attempts to pharmacologically target and exploit this system have been challenging.

Recent reports identified an interaction of chemokine receptors with receptor activity-modifying proteins (RAMPs). The three members (RAMP1-3) of this single-pass transmembrane protein family were initially found to form heterodimers with some class B GPCRs (including *e.g.* the calcitonin receptor, *CTR*, and the calcitonin receptor-like receptor, *CLR*), resulting in pharmacological modulation of various stages of these receptors’ life cycle, including membrane trafficking and ligand preference (10, 11).

The list of suggested RAMP-interacting GPCRs is steadily growing and now includes receptors from most human classes of GPCRs (12). Functionally, RAMPs have been shown to affect various members of class B1 (including *e.g.* CLR, CTR, GIPR, GLP1R, glucagon receptor, (10, 13, 14, 15)), class A (MRGPRX & ACKR3) (16, 17), and class C (CASR) (18) GPCRs (reviewed in (12)). Notably, the functional effects of RAMPs are highly versatile, resulting in a variety of different functional outcomes depending on the specific RAMP:GPCR pairing (reviewed in (19)) and, despite high-resolution structures of GPCR: RAMP complexes (20, 21, 22, 23), the mechanisms involved in RAMP regulation are not well understood. In addition, for most GPCRs, the functional effects of RAMPs remain uncharacterized, and more studies are needed to establish the impact of RAMPs on the large number of suggested RAMP-interacting receptors.

RAMP3 has been shown to increase the ability of ACKR3 to scavenge ligands by promoting its recycling to the plasma membrane in the presence of the N-ethylmaleimide-sensitive factor (NSF). In addition, deletion of either ACKR3 or RAMP3 abolished retinal angiogenesis in mice, highlighting the significance of this interaction *in vivo* (17). Meanwhile, the interaction of RAMPs with CXCR4 was identified from protein-protein interaction studies (17) but not explored further to this point.

Here, we characterized the functional effects of RAMPs on ACKR3 and CXCR4 chosen based on their shared ligand, CXCL12. We show that RAMP3 attenuates ACKR3 ligand-induced internalization and ligand scavenging without effects on CXCR4 endocytosis or G protein signaling. We further show that RAMPs impair constitutive and ligand-induced β-arrestin recruitment to both CXCR4 and ACKR3 and that this modulation occurs independent of ACKR3’s N-terminus. This elucidation will provide novel and valuable insights into regulatory mechanisms around the CXCL12 signaling axis.

## Results

### Surface expression of CXCR4 and ACKR3 is unaltered by RAMPs

Since RAMPs are involved in the trafficking of several GPCRs to the cell membrane (11), we first set out to determine whether the co-expression with RAMPs alters ACKR3 and CXCR4 surface expression. For this, we employed SNAP-surface labeling of receptors as previously described (24). The SNAP-receptor sequence was preceded by an N-terminal signal sequence (IL2Rα 1–21) to improve membrane localization of receptors and a FLAG-tag (DYKDDDDK) to allow for immunoblotting. RAMP constructs were preceded by a 3x Human influenza hemagglutinin (HA)-tag (HA-RAMP).

**Figure 1:**
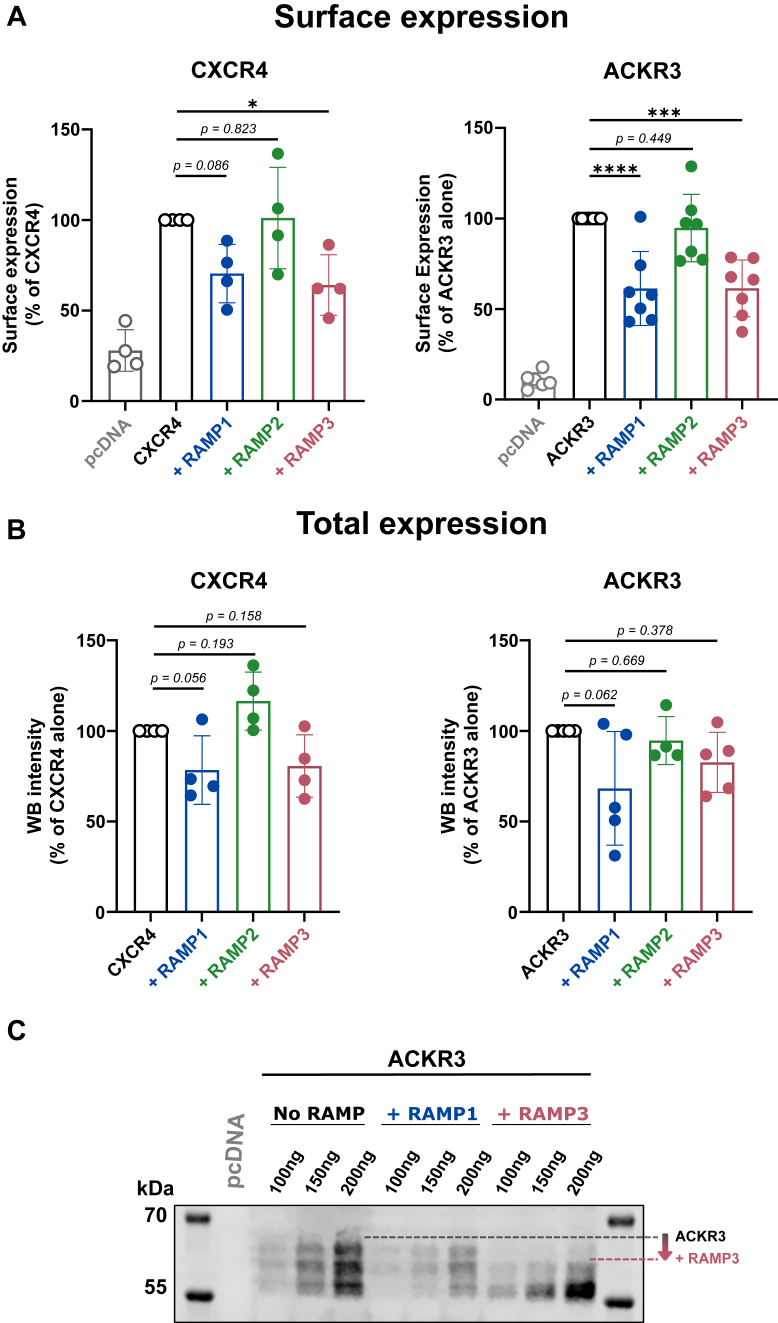
**CXCR4 and ACKR3 expression.***A*, surface expression of both CXCR4 and ACKR3 was quantified by SNAP-labeling in HEK293A cells transfected with either receptor −/+ RAMP1-3 (n = 4 for CXCR4 and n = 7 for ACKR3). *B*, total expression of CXCR4 and ACKR3 was quantified by Western blot analysis of transfected cells. (n = 4 for CXCR4 and n = 5 for ACKR3). (theoretical MWs: SNAP-ACKR3 62.355 kDa, SNAP-CXCR4 60.608 kDa). Each data point represents one experiment shown as a percentage of the control column (receptor alone) and plotted as mean ± SD (bar charts) (*A* & *B*). *C*, Western blot of ACKR3 (100–200ng transfected ACKR3 DNA + RAMP3 in 1:3 receptor:RAMP ratio) from transiently transfected, solubilized HEK293A cells. Statistical analysis was performed on the non-normalized data using ordinary two-way ANOVA of main effects with Dunnett’s test for multiple comparisons (*A* & *B*) (∗∗∗∗*p* < 0.0001, ∗∗∗*p* < 0.001, ∗*p* < 0.05).

We found that HA-RAMP2 does not affect either receptor’s surface availability, while HA-RAMP1 and HA-RAMP3 co-expression diminishes surface levels of both ACKR3 and CXCR4 by ∼40% compared to the receptor alone (Fig. 1*A*). To determine whether this reduction is caused by lower expression, we applied immunoblotting to quantify total receptor expression in the transfected cells (Fig. S1). We found that co-expression with both HA-RAMP1 and HA-RAMP3 reduces total receptor expression to a similar extent as the reduction of surface expression (Fig. 1*B*). This indicates that RAMPs do not alter the cellular distribution of CXCR4 or ACKR3 but that the lower surface availability is an effect of reduced total expression.

Immunoblotting further revealed that both ACKR3 and CXCR4 are N-glycosylated (as predicted based on NxS/T motifs), evident from a downward shift of the band after treatment with Peptide:N-glycosidase F (PNGase F) to remove N-linked glycosylation (Fig. S1). ACKR3 exhibits a diverse glycosylation pattern, which manifests as a blurry band on the Western blot and CXCR4 glycosylation appears more uniform across the cell. This is likely due to ACKR3 having three N-terminal asparagine residues (N13, N22, N39; Uniprot) predicted to be N-glycosylated, while CXCR4 is (mainly) glycosylated at one residue (N11) (25, 26). In the presence of HA-RAMP3, ACKR3 is shifted toward a less-glycosylated state (Fig. 1*C*). This could mark an effect of RAMP3 on ACKR3 maturation and calls for further studies of the effects of RAMPs on GPCR glycosylation.

The functionality of the used HA-RAMP constructs was confirmed by increased CLR surface expression upon the addition of each RAMP (11) as quantified by SNAP-surface labeling (Fig. S2*A*). When co-expressed with ACKR3, all HA-RAMP constructs are detectable by immunoblotting but HA-RAMP1 and HA-RAMP3 are expressed at significantly higher levels than HA-RAMP2 (Fig. S2*B*). In accordance with previous studies (27), treatment with PNGase F confirmed that RAMP1 is not N-glycosylated, as opposed to RAMP2 and RAMP3 (RAMP2<RAMP3). Flow cytometry further revealed that HA-RAMP3 is present at the cell surface (by itself and with ACKR3), whereas HA-RAMP1 and HA-RAMP2 do not reach the cell surface when co-expressed with ACKR3 (Fig. S2*C*), similarly as previously described (17).

In summary, RAMPs do not alter the cellular localization of either ACKR3 or CXCR4, and only RAMP3 reaches the cell surface regardless of receptor co-expression. Furthermore, both ACKR3 and CXCR4 are glycosylated, and ACKR3 glycosylation is reduced in the presence of RAMP3.

### RAMP3 decreases CXCL12-induced internalization of ACKR3 but not CXCR4

**Figure 2:**
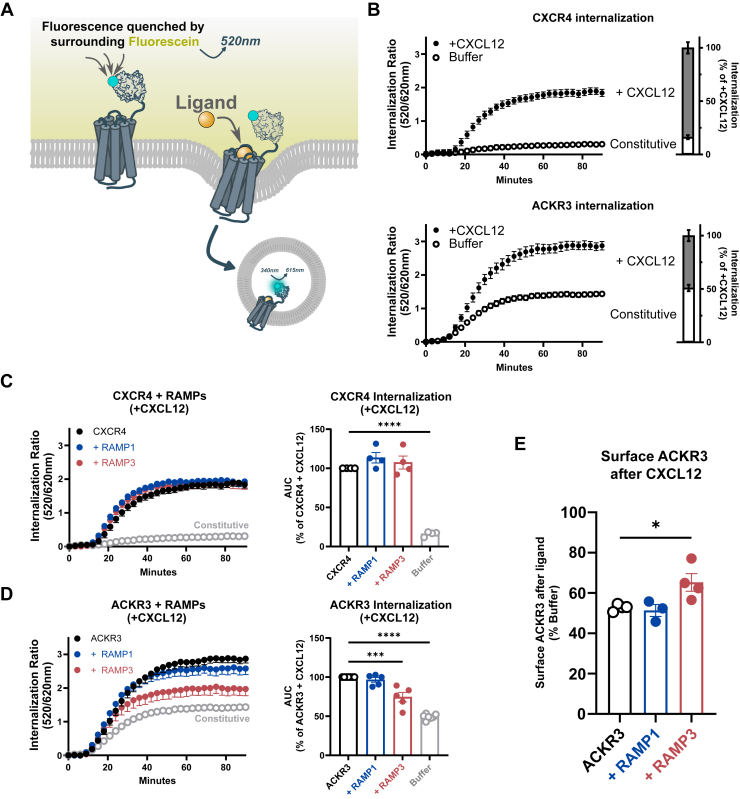
**Constitutive and CXCL12-induced receptor internalization in the presence of RAMPs.***A*, schematic representation of the internalization assay. SNAP-receptors are labeled at the cell surface and internalization is stimulated with ligand (CXCL12). When receptors internalize, the quenching effects of the extracellularly surrounding Fluorescein are lost, resulting in a measurable increase of 615nm emission, consistent with receptor endocytosis. *B*, constitutive and CXCL12-internalization of CXCR4 and ACKR3), shown as mean ± SEM of independent experiment (n = 4 for CXCR4 and n = 7–10 for ACKR3). The bar plot illustrates the percentage of constitutive internalization as compared to CXCL12-induced internalization for either receptor, quantified as the AUC over 90 min (mean ± SEM). *C* and *D*, CXCL12-induced internalization of CXCR4 (*C*) or ACKR3 (*D*) in the presence of RAMPs. Internalization curves (*left*) are presented as mean ± SEM of n = 4 and n = 5 to 10 experiments for CXCR4 and ACKR3, respectively. For the quantification of internalization (bar plots, *right*), each data point represents the AUC (90 min) of one experiment shown as a percentage of the control column (receptor alone) and plotted as mean ± SEM of biological replicates (n = 4 and n = 5–10 experiments for CXCR4 and ACKR3, respectively). *E*, remaining SNAP-ACKR3 at the cell surface after 30-min incubation with CXCL12, plotted as a percentage of the remaining receptor after treatment with buffer (basal conditions) (n = 3–4). All experiments were performed in technical triplicates. Statistical analysis was performed on the non-normalized data using ordinary two-way ANOVA of main effects with Dunnett’s test for multiple comparisons and presented as percentage of receptor alone (mean ± SEM) (∗∗∗∗*p* < 0.0001, ∗∗∗*p* < 0.001, ∗∗*p* < 0.01, ∗*p* < 0.05, - = ns).

Internalization of CXCR4 and other GPCRs desensitizes signaling by reducing the number of receptors in the cell membrane. In addition, ACKR3’s physiological function is largely ascribed to the scavenging of extracellular ligands through internalization and subsequent degradation. We, therefore, investigated the functional effects of RAMPs on ACKR3 and CXCR4 internalization by applying a previously described diffusion-enhanced resonance energy transfer (DERET) based internalization assay, where internalization of labeled SNAP-receptor is quantified by an increase in donor/acceptor ratio (Fig. 2*A*) (24).

When tested with CLR, only HA-RAMP1 and HA-RAMP3 co-expression led to receptor internalization upon stimulation with its endogenous ligand adrenomedullin, but HA-RAMP2 did not (Fig. S2*D*). This contradicts previous reports that the CLR:RAMP2 can, in fact, internalize upon adrenomedullin-stimulation (28, 29), suggesting that HA-RAMP2 is not functional or that its poor expression does not allow for sufficient CLR: RAMP2 complex formation that would result in measurable internalization. For this reason, we omitted the HA-RAMP2 construct from the internalization experiments with CXCR4 and ACKR3.

Supporting previous reports (24, 30, 31), CXCR4 exhibits very limited constitutive internalization but is strongly endocytosed when activated by its endogenous agonist CXCL12. On the other hand, ACKR3 internalization occurs constitutively and is boosted when stimulated by CXCL12 (Fig. 2*B*). Upon co-expression with a dominant negative dynamin mutant (Dyn-K44A), which is unable to bind and hydrolyze GTP and therefore inhibits endocytosis (32) (Fig. S3*A*), ACKR3 surface expression increases and approaches that of CXCR4 (Fig. S3*B*), confirming that ACKR3 and CXCR4 are expressed at similar levels and further supporting that ACKR3 is constitutively internalizing, resulting in a large fraction of ACKR3 being located intracellularly.

CXCR4’s low degree of constitutive endocytosis was unaffected by RAMPs (Fig. S4*A*), just as ACKR3’s constitutive internalization was not affected in the presence of RAMPs (Fig. S4*B*), consistent with the finding that RAMPs don’t affect ACKR3’s cellular localization (Fig. 1). When stimulated with CXCL12, CXCR4 internalization was unaltered by the addition of RAMPs (Fig. 2*C*). However, HA-RAMP3 reduced total ACKR3 internalization by ∼25% in response to CXCL12, compared to ACKR3 alone, whereas HA-RAMP1 exerted no significant effects (Figs. 2*D* and S5). Seeing that we only observe this reduction of ligand-induced but not constitutive internalization, RAMP3 attenuates the purely ligand-induced internalization by ∼50%.

ACKR3 internalization is independent of surface expression in the given expression range (Fig. S6), which confirms that the reduced ACKR3 internalization is specifically caused by RAMP3 and not the decrease in total receptor expression (Fig. 1*B*). To further validate this, we quantified ACKR3 surface availability before and after CXCL12 stimulation, both in the presence and absence of HA-RAMP3. After CXCL12-induced internalization of ACKR3, ∼53% of ACKR3’s surface pool remained at the surface, whereas ∼65% of ACKR3 remained at the surface when HA-RAMP3 was present. In contrast, HA-RAMP1 did not have any effects on the remaining surface pool of ACKR3 after CXCL12 stimulation (Fig. 2*E*), further supporting that RAMP3 reduces ACKR3 internalization in response to CXCL12 stimulation.

Taken together, these data show that RAMPs do not affect constitutive internalization of either ACKR3 or CXCR4, but that RAMP3 inhibits ACKR3’s, but not CXCR4’s ability to endocytose upon agonist activation.

### RAMP3 impairs CXCL12 scavenging by ACKR3

**Figure 3:**
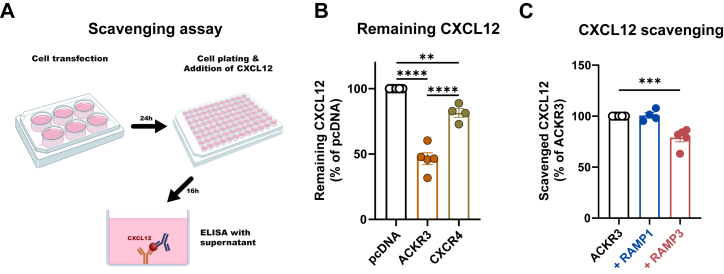
**CXCL12 scavenging determined by ELISA.***A*, schematic representation of the scavenging assay: Cells were transfected with receptor, receptor + RAMPs or pcDNA and plated in a 96-well plate. Following overnight incubation with CXCL12, CXCL12 concentration in supernatants was determined by ELISA. *B*, remaining CXCL12 in cell supernatants in the absence or presence of either ACKR3 or CXCR4, presented as percentage of pcDNA (n = 4) *C*, CXCL12 scavenged by ACKR3 in the presence or absence of either RAMP1 or RAMP3 (n = 4–5). All experiments were performed in technical triplicates. Statistical analysis was performed on the non-normalized data using ordinary two-way ANOVA of main effects with Dunnett’s test for multiple comparisons and presented as percentage of the control column (pcDNA (*B*) or ACKR3 (*C*)) (mean ± SEM) (∗∗∗∗*p* < 0.0001, ∗∗∗*p* < 0.001, ∗∗*p* < 0.01, - = n.s.).

As ACKR3 is an atypical receptor with ligand scavenging being arguably its most important physiological role, we wanted to assess whether the observed effects of RAMP3 on ligand-induced internalization are reflected in a reduced ability of ACKR3 to scavenge CXCL12 from the extracellular milieu. We therefore quantified the removal of extracellular CXCL12 by ELISA (Fig. 3*A*) and found that, as expected, ACKR3 is significantly more efficient in scavenging CXCL12 from the extracellular medium than CXCR4 (Fig. 3*B*). Furthermore, ACKR3’s scavenging ability was markedly decreased in the presence of HA-RAMP3, but unaffected by HA-RAMP1 (Fig. 3*C*). This is in line with our previous observations that RAMP3 reduces ligand-induced receptor endocytosis, consequently resulting in less scavenging of ligands. Hence, by inhibiting the ligand-induced pathway of ACKR3 internalization, RAMP3 negatively modulates ACKR3’s scavenging function.

### RAMP3 affects ACKR3 endocytosis in a ligand-independent manner

**Figure 4:**
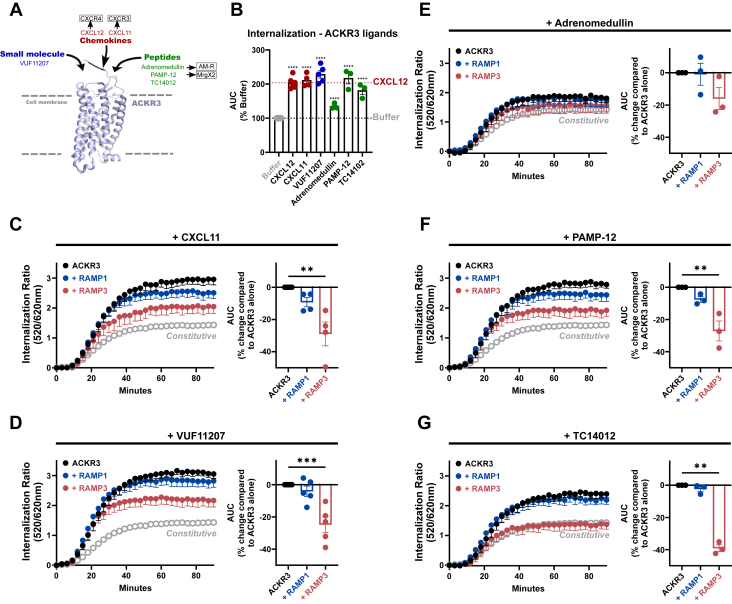
**ACKR3 internalization (−/+ RAMPs) in response to different agonists.***A*, ACKR3 agonists are grouped into chemokines (*red*), small molecule (*blue*), and peptides (*green*) (PDB: 7SK6). *B*, AUC (90 min) analysis of ACKR3 internalization curves with the different ACKR3 agonists, presented as percentage of constutitive internalization (n = 3–10). *C*–*H*, internalization of ACKR3 (−/+ RAMP1&3) in response to the chemokine CXCL11 (n = 4) (*C*), the small molecule VUF11207 (n = 5) (*D*), and the peptides adrenomedullin (n = 3) (*E*), PAMP-12 (n = 3) (*F*), and TC14012 (n = 3) (*G*). Internalization curves (*left* in each panel) are mean ± SEM of biological replicates and quantified as the AUC over 90 min (barplots, *right* in each panel), where each datapoint represents the mean of a single experiment. All experiments were performed in technical triplicates. Statistical analysis was performed on the non-normalized data by ordinary two-way ANOVA of main effects with Dunnett’s correction for multiple testing. (∗∗∗∗*p* < 0.0001, ∗∗∗*p* < 0.001, ∗∗*p* < 0.01, ∗*p* < 0.05, - = ns).

To assess whether ACKR3’s reduced endocytosis in presence of RAMP3 is specific to the agonist CXCL12, we tested whether ACKR3 would be similarly affected when activated by different ligands, including the chemokine agonist CXCL11, the small synthetic agonist VUF11207 (33), as well as the peptides adrenomedullin, proadrenomedullin N-terminal 20 peptide (9, 10, 11, 12, 13, 14, 15, 16, 17, 18, 19, 20) (PAMP12, an adrenomedullin-derived peptide ACKR3 agonist (7)), and TC14012 (a synthetic peptide agonist (34, 35)) (Fig. 4*A*).

We found that at saturating concentrations, all tested ligands induce internalization of ACKR3 to a similar degree as CXCL12. The exception is adrenomedullin stimulation, which results in internalization only to a small extent even at high, however non-saturating concentrations (10μM) (7, 17) (Fig. 4*B*). This would be in line with reports that ACKR3 does not scavenge adrenomedullin and that CXCL12 uptake is not competitively diminished by high concentrations of adrenomedullin (10μM) (36). We cannot, however, exclude the possibility that this finding is due to the previously described low potency of adrenomedullin at ACKR3 (7).

In the presence of HA-RAMP3, ACKR3 internalization was reduced in response to all ligands (Fig. 4, *C*–*G*), just as observed for CXCL12 (Fig. 2*D*). However, in the case of the synthetic peptide TC14012, ligand-induced internalization is entirely abolished and reduced to the level of constitutive internalization (Fig. 4*G*), potentially relating to its somewhat unique binding mode at ACKR3, which is similar to that of the antagonist CVX15 at CXCR4 (37).

The finding that RAMP3 negatively affects ACKR3 internalization is consistent throughout protein, peptide, and small molecule ligands indicating that this occurs in response to all ligands, with different ligands affected to different extents. Furthermore, the small-molecule agonist VUF11207 binds ACKR3 only in its orthosteric pocket, indicating that RAMP3:ligand interactions are not involved in these functional effects.

### RAMP3 mitigates but does not prevent ACKR3 internalization in response to ligand stimulation

**Figure 5:**
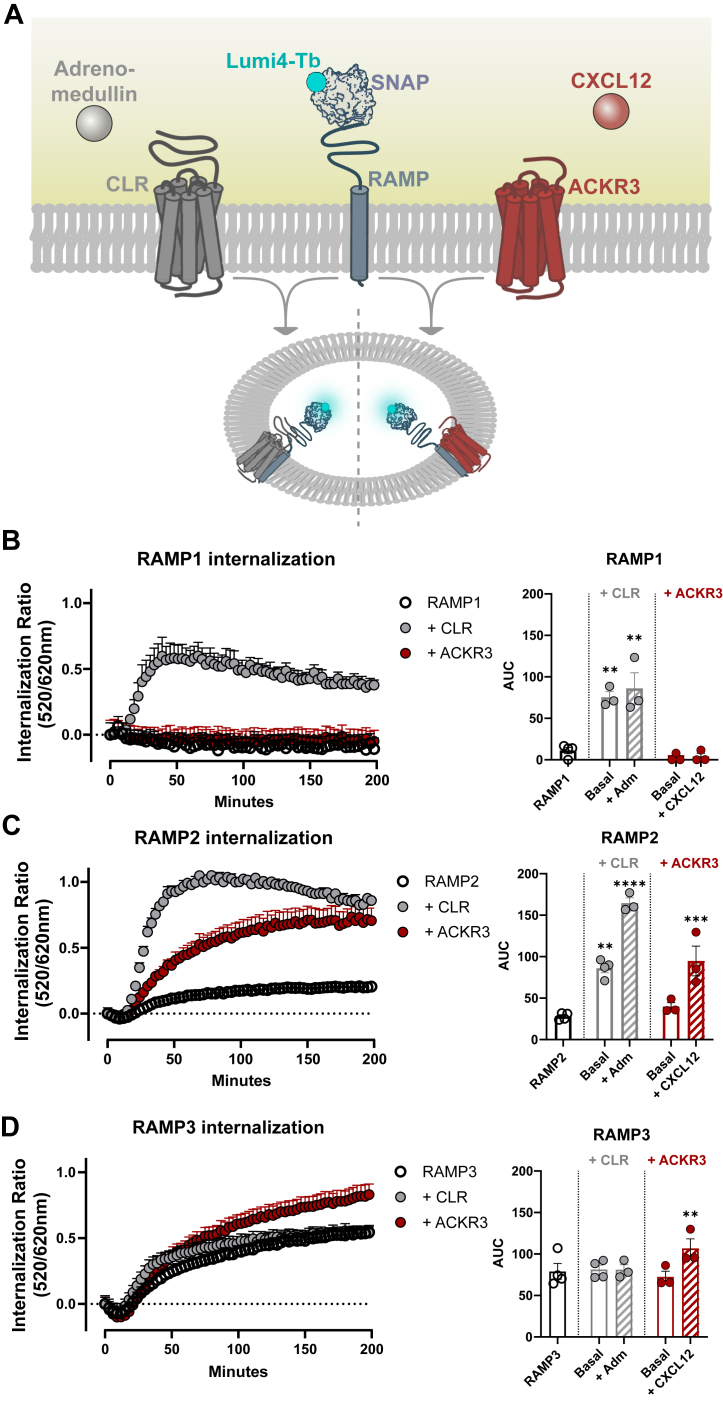
**Internalization of SNAP-RAMPs.***A*, schematic representation of the internalization assay: SNAP-RAMPs are co-expressed with CLR (*grey*) or ACKR3 (*red*) and RAMP internalization is monitored over time, as described in Fig. 2. *B*–*D*, internalization curves (*left* in each panel) for RAMP alone (*open circles*) and co-expressed with CLR (*gray circles*) or ACKR3 (*red circle*s). In the presence of a receptor, RAMP internalization was stimulated with receptor-specific ligands (adrenomedullin for CLR, CXCL12 for ACKR3). Internalization curves are shown as mean ± SEM of 3 to 4 independent experiments (n = 3–4). AUC of internalization curves (200 min) analyzed for RAMP1 (*B*), RAMP2 (*C*), and RAMP3 (*D*) in bar plots (*right*), both in the presence and in the absence of receptor and buffer or respective ligand adrenomedullin (CLR) or CXCL12 (ACKR3). All experiments were performed in technical triplicates. Statistical analysis was performed on the non-normalized data by ordinary two-way ANOVA of main effects with Dunnett’s correction for multiple testing. (∗∗∗∗*p* < 0.0001, ∗∗∗*p* < 0.001, ∗∗*p* < 0.01,- = ns).

Since RAMP3 inhibits the ligand-induced internalization of ACKR3, our next objective was to determine if RAMP3 binding diminishes or completely prevents receptor internalization. To test this, we monitored the CXCL12-induced internalization of SNAP-tagged (ST-)RAMPs in the presence and absence of receptors. If the ST-RAMP internalizes in the presence of a receptor and when stimulated with the corresponding ligand, this is indicative of internalization of the receptor:RAMP complex (Fig. 5*A*). It should be noted that, like the ST-receptor constructs before, the ST-RAMP constructs utilized here are preceded by an exogenous signal sequence to aid membrane localization.

We found that ST-RAMP1 was surface expressed in low amounts in the absence of co-expressed receptor (Fig. S7*A*), virtually indistinguishable from the vector control, whereas ST-RAMP2 and ST-RAMP3 reached the surface in high amounts (RAMP2<RAMP3). Immunoblotting revealed that the decreased surface availability of RAMP1 is not due to total RAMP1 expression (Fig. S7*B*), but to RAMP1 not being able to efficiently reach the cell surface by itself, as reported before (15, 17, 27), regardless of the exogenous signal sequence. Consequently, RAMP1 shows no signs of internalization in the absence of a co-expressed receptor (Fig. 5*B*), whereas RAMP2 and RAMP3 exhibit different degrees of constitutive internalization (Fig. 5, *C* and *D*, RAMP2<RAMP3), likely by co-internalization with, *e.g.*, GPCRs endogenously expressed in Human embryonic kidney-293 (HEK293) cells (38).

Upon co-expression with CLR, there is a trend towards slightly increased RAMP1 surface expression (Fig. S7*C*), consistent with RAMP1 and CLR chaperoning each other to the cell surface. As expected, the CLR:RAMP1 (Fig. 5*B*) and CLR:RAMP2 (Fig. 5*C*) complexes internalize both constitutively and when stimulated with adrenomedullin, in accordance with previous studies (28). Surprisingly, RAMP3 does not internalize when co-expressed with CLR and stimulated with adrenomedullin (Fig. 5*C*), possibly due to a combination of the low total internalization of CLR:RAMP3 (29) and the relatively high constitutive internalization of RAMP3 observed here.

When co-expressed with ACKR3, RAMP1 showed no signs of internalization (Fig. 5*B*), most likely suggesting that there is no interaction between the two due to RAMP1 not reaching the cell surface. However, both RAMP2 and RAMP3 showed increased internalization when co-expressed with ACKR3 and stimulated with CXCL12 (Fig. 5, *C* and *D*), which suggests they are both able to bind ACKR3 when present at the cell membrane.

Taken together with our finding that RAMP3 reduces ACKR3 internalization (Fig. 2, *D* and *E*), this suggests that RAMP3 makes the receptor less likely to be internalized after binding to CXCL12, rather than fully preventing its internalization.

### Constitutive and ligand-induced β-Arrestin recruitment to ACKR3 and CXCR4 is impeded by RAMP3

**Figure 6:**
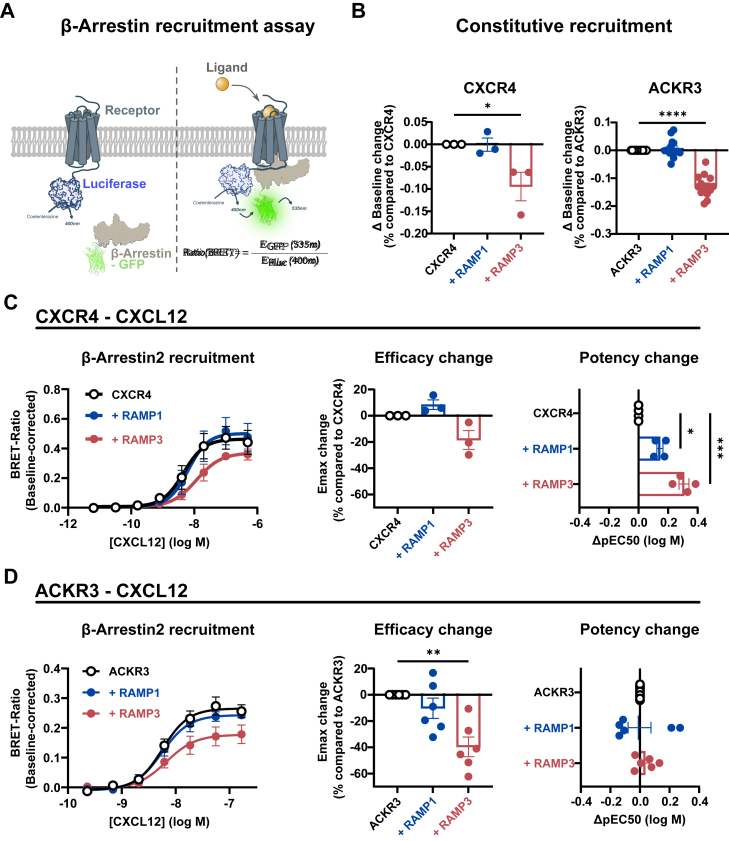
β**-Arrestin recruitment to CXCR4 and ACKR3 −/+ RAMPs.***A*, schematic representation of the assay. Upon recruitment of GFP- β-arrestin to the receptor-Rluc, energy is transferred (BRET) from the Rluc (donor) to the GFP (acceptor), resulting in a measurable increase of the BRET ratio. *B*, constitutive β-Arrestin recruitment to CXCR4 (*top*, n = 3) and ACKR3 (*bottom*, n = 14), presented as the decrease in baseline of BRET-ratios compared to receptor alone (Δ Baseline change) *C* and *D*, CXCL12-induced recruitment of β-Arrestin to CXCR4 (n = 4) (*C*) and ACKR3 (n = 6) (*D*) (−/+ RAMPs) (*left*), with potency differences (ΔpEC50, pEC_50_(_+RAMP)_ – pEC_50(only receptor)_) compared to receptor alone (bar graph, *middle*) and efficacy changes (% Emax change) (bar graph, *right*) in absence and presence of RAMPs. Dose-response curves (*left*) are presented as mean ± SEM of independent experiments and potency and efficacy changes from each experiment plotted as a single data point (mean ± SEM). All experiments were performed in technical triplicates. Statistical analysis was performed on the non-normalized data by ordinary two-way ANOVA of main effects with Dunnett’s correction for multiple testing. (∗∗∗∗*p* < 0.0001, ∗∗∗*p* < 0.001, ∗∗*p* < 0.01, ∗*p* < 0.05, - = ns).

While only CXCR4 couples to G proteins, both CXCR4 and ACKR3 recruit β-arrestin upon activation. β -arrestin recruitment desensitizes CXCR4 and modulates G-protein coupled signaling pathways. In the case of ACKR3, the exact role of β-arrestin coupling is unclear to date, as it seems dispensable for the receptor’s internalization (4, 9, 39, 40, 41). We found that RAMPs did not modulate Gi-mediated cAMP inhibition through CXCR4 after five or 30 min (Fig. S8). Given that RAMP3 attenuated ACKR3 internalization in response to ligand binding, we next determined the effects of RAMPs on β-arrestin recruitment to ACKR3 and CXCR4 following stimulation by CXCL12 (Fig. 6*A*).

Interestingly, although CXCR4 internalization is unaffected by the presence of RAMPs, we observed that RAMP3 reduced the constitutive recruitment of β-arrestin to CXCR4, as visible from a decrease in the baseline (Fig. 6*B*). Similarly, RAMP3 reduced the efficacy (E_max_) of β-arrestin recruitment to CXCR4 by 18.5% with a slight decrease in potency, more than that observed with RAMP1 (Fig. 6*C*).

Even though ACKR3’s basal internalization (= in the absence of ligand) was unaffected by RAMP3, we found that RAMP3 reduced the constitutive recruitment of β-arrestin to ACKR3 (Fig. 6*B*). Moreover, co-expression with RAMP3 decreased the CXCL12-induced Emax of recruitment by ∼40% (Fig. 6*D*), similarly but to a greater extent than observed for CXCR4. We confirmed that RAMP3 did not affect the total expression of either receptor by quantification of Rluc-signal after substrate addition (Fig. S9).

Of note, CXCR4 has higher BRET values than ACKR3. However, BRET values may be affected by different lengths of ACKR3 and CXCR4 C-termini leading to different probe locations and/or different arrestin binding modes to the two receptors, which may explain the apparent contrast with recent studies suggesting that ACKR3 recruits more arrestin than CXCR4 (3).

Additionally, RAMP1 and RAMP3 reduced arrestin recruitment in an indirect (bystander) BRET-based approach; however, this reduction can likely at least in part be explained by the decreased ACKR3 surface availability upon co-expression with RAMP3 and, to a greater extent, RAMP1 (Fig. S10).

Based on the presented findings, we conclude that RAMP3 reduces both constitutive and ligand-induced β-arrestin recruitment to ACKR3 and CXCR4 when stimulated with CXCL12.

### Potency of β-Arrestin recruitment to ACKR3 is partially ligand-dependent, without interactions with receptor N-terminus

We next asked whether β-arrestin recruitment induced by ACKR3’s other agonists is similarly affected by RAMP3.

**Figure 7:**
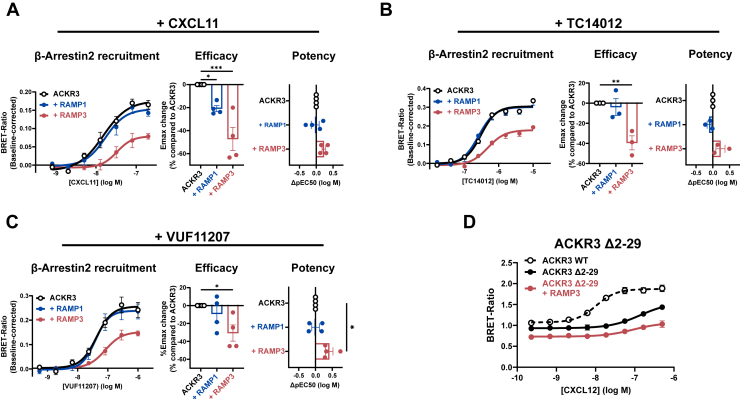
**β-Arrestin recruitment to ACKR3 (−/+ RAMPs) with different ligands.***A*–*C*, Arrestin recruitment was induced by CXCL11 (n = 4) (*A*), TC14012 (n = 3) (*B*) and VUF11207 (n = 4) (*C*). Efficacy changes are presented as % change of Emax of ACKR3 alone (bargraph, *middle* in each panel) and potency differences as ΔpEC50 (pEC_50_(_+RAMP)_ – pEC_50(only receptor)_) compared to ACKR3 alone (bargraph, *right* in each panel). *D*, β-Arrestin recruitment to an N-terminal cleavage mutant of ACKR3 (−/+ RAMP3), where 28 residues have been removed from ACKR3’s N-terminus (n = 3). Dose-response curves are presented as mean ± SEM of independent experiments preformed in technical triplicates. Statistical analysis was performed on the non-normalized data by ordinary two-way ANOVA of main effects with Dunnett’s correction for multiple testing. (∗∗∗∗*p* < 0.0001, ∗∗∗*p* < 0.001, ∗∗*p* < 0.01, ∗*p* < 0.05, - = ns).

CXCL11, VUF11207, and TC14012 induced β-arrestin recruitment to ACKR3 with similar potencies as described previously (35, 37, 42, 43). In the presence of HA-RAMP3, the efficacy of β-arrestin recruitment is similarly affected as observed for CXCL12 (Fig. 6*D*), with a decrease of ∼47%, ∼30%, and ∼39% for CXCL11, VUF11207, and TC14012, respectively (Fig. 7, *A*–*C*). Interestingly, while CXCL11 and TC14012 potency remains virtually unchanged in the presence of HA-RAMP3, VUF11207-induced β-arrestin recruitment to ACKR3 is less potent (pEC50 7.39 without RAMP vs 6.98 with HA-RAMP3, *p* = 0.014). PAMP12 and adrenomedullin potency in the β-arrestin recruitment was too low to reliably fit the dose-response curves (Fig. S11).

In summary, the effect of RAMP3 on ACKR3’s β-arrestin recruitment was visible for all ligands tested, albeit with slight effects on the potency of the small molecule VUF11207. This indicates that there are slight differences in how different ligands are affected by RAMP3, but that inhibition of β-arrestin recruitment to ACKR3 by RAMP3 occurs regardless of ligand. This further supports the notion that the complex formation is limited to ACKR3:RAMP3 interactions, rather than RAMP3 engaging in interactions with ligands.

Moreover, given the crucial involvement of the large extracellular domain of class B GPCRs in their interaction with RAMPs (11), we hypothesized that RAMP3 exerts some of its effects by engaging in similar interactions with ACKR3’s N-terminus. We tested this using a N-terminally cleaved mutant of ACKR3, where 28 residues have been removed from the N-terminus (Δ2-29). As previously reported (44), the removal of the N-terminus of ACKR3 severely impacted CXCL12-induced β-arrestin recruitment, with a major reduction of potency (Fig. 7*D*). Strikingly, the effects of RAMP3 remained intact, *i.e.* a reduced baseline and efficacy of β-arrestin recruitment. This shows that the N-terminal 28 residues are dispensable for RAMP3’s modulation of ACKR3 and likely not involved in interactions with RAMP3.

### RAMPs modulate ACKR3 function at the cell membrane

Recent research has shown that GPCRs can not only initiate signaling when located at the cell surface but continue to do so from intracellular compartments, *e.g.* after ligand-induced internalization (45). To assess whether the observed RAMP effects are due to complex formation at the cell membrane, we utilized the previously mentioned Dyn-K44A to inhibit endocytosis.

**Figure 8:**
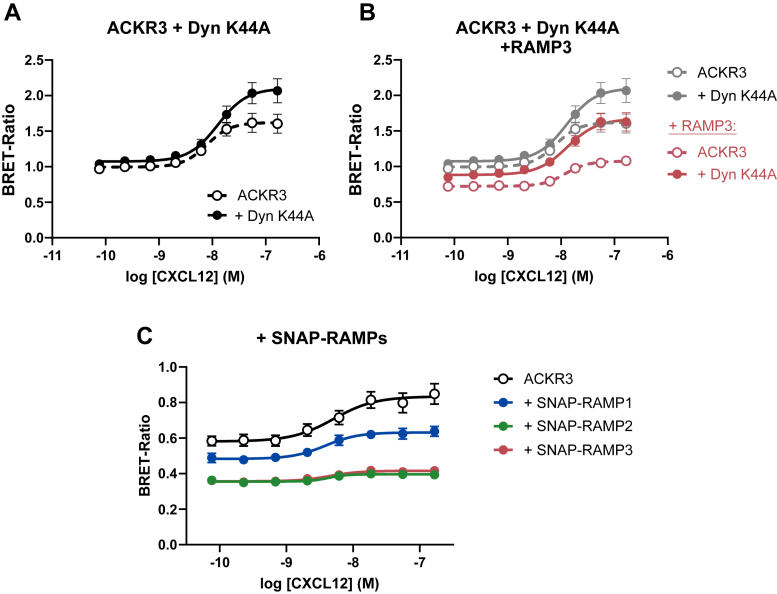
**β-Arrestin recruitment to surface ACKR3.***A*, ACKR3 was co-expressed with the dominant negative Dynamin mutant (Dyn-K44A) to retain the receptor at the cell surface, and β-arrestin2 recruitment was quantified at different CXCL12 concentrations (n = 4). *B*, CXCL12-induced β-arrestin recruitment to surface-retained ACKR3 in the presence of RAMP3 (n = 4). *C*, β-arrestin2 recruitment to ACKR3 in the presence or absence of SNAP-RAMPs and induced with CXCL12 (n = 3). All dose responses are shown as mean ± SEM of independent experiments performed in technical duplicates.

ACKR3 internalization was effectively inhibited by co-expression with Dyn-K44A (32) in a dose-dependent manner (Fig. S3*B*), as previously observed for other GPCRs (46, 47, 48) (reviewed in (49)). Upon co-expression with Dyn-K44A and retainment of the ACKR3 at the cell membrane, efficacy (E_max_) of β-arrestin recruitment to the receptor was increased (Fig. 8*A*), suggesting that ACKR3- β-arrestin coupling preferentially occurs at the cell surface. When retained at the cell surface and in the presence of HA-RAMP3, the previously observed decrease of E_max_ and baseline is visible to a similar degree (Fig. 8*B*), suggesting that RAMP3 exerts its effects on ACKR3 at the cell membrane.

Taken together, our results show RAMP3 inhibits ACKR3 internalization and β-arrestin recruitment by regulating the receptor at the plasma membrane. However, unlike CLR, ACKR3 does not chaperone RAMPs to the plasma membrane, and RAMP3 is the only isoform that reaches the cell surface in a receptor-independent manner. Therefore, it is unclear if the other RAMPs affect ACKR3 when present in the same compartment. To test this, we again utilized the ST-RAMP constructs, where ST-RAMP2 and, to a lesser extent, ST-RAMP1 reach the plasma membrane when co-expressed with ACKR3. All three ST-RAMP reduce both constitutive and CXCL12-induced β-arrestin recruitment to ACKR3, similar to the HA-RAMP3 construct (Fig. 8*C*). This shows that when present in the same membrane, all three RAMP isoforms regulate ACKR3 function. Furthermore, this suggests that the ability of RAMP3 to regulate receptor function in the absence of an exogenous membrane localization signal is linked to its receptor-independent trafficking to the cell membrane.

## Discussion

Recent reports show that RAMPs interact with receptors from all GPCR classes, and the regulation of GPCRs by RAMPs is gaining increasingly more interest in the field. To this date, RAMPs have been shown to affect a plethora of GPCRs, especially members of class B and more recently also class A (12, 16, 17, 50). Chemokine receptors are a subgroup of class A GPCRs, and multiple members, including CXCR4 and ACKR3, interact with RAMPs (17, 50).

Here, we contributed to the functional understanding of RAMP interactions by characterizing the effects of RAMPs on signaling and endocytosis of the chemokine receptors CXCR4 and ACKR3. The signaling axis around CXCL12, CXCR4, and ACKR3 plays a major role in a plethora of pathological, immunological, and developmental processes and is indispensable for survival (1, 2). While CXCR4 signals through Gi, resulting in cell proliferation, differentiation, and migration, ACKR3 regulates CXCR4 activation by scavenging and removal of CXCL12 from the extracellular milieu. Owing to ACKR3’s ligand variety, similar effects have been proposed on other GPCRs, including CXCR3, opioid receptors, adrenomedullin receptors and others (5, 7). Understanding the functional modulation of ACKR3 is, therefore, not only relevant in the context of CXCL12 but also in other systems.

While RAMP3 has previously been shown to facilitate ACKR3 recycling after ligand exposure, we observed that RAMP3, but not RAMP1 can negatively modulate ACKR3’s ligand-induced internalization and, consequently, its physiologically relevant scavenging properties. This challenges the notion that the ACKR3:RAMP interaction is necessary for efficient chemokine scavenging, as proposed previously (17). Furthermore, while ACKR3 endocytosis occurs independently of β-arrestin (39, 40, 41)), recruitment of β-arrestin to ACKR3 was similarly affected by RAMP3. Interestingly, only ligand-induced, but not constitutive internalization was altered by RAMP3, whereas both basal and ligand-induced recruitment of β-arrestin was diminished in the presence of RAMP3. Some GPCRs utilize distinct mechanisms for constitutive and ligand-induced internalization (including *e.g.* β2AR, M3R & CXCR4, reviewed in (49)) and are potentially the underlying phenomenon for part of these observations. Notably, the effects of RAMP3 on ACKR3 function observed in this study closely resemble the effects observed upon knock-out of G protein-coupled receptor kinase 5 (GRK5), including the reduced β-arrestin recruitment and ligand-induced internalization, all the while maintaining constitutive internalization (40). Together, these findings could suggest that RAMP3 interferes with ACKR3 phosphorylation by GRKs, but more research is needed to validate this.

RAMPs do not affect the coupling of CXCR4 to G proteins but RAMP3 reduces β-arrestin recruitment to the receptor. The observed effects on both CXCR4 and ACKR3 suggest that RAMPs might, in fact, elongate the duration of CXCL12 signaling at CXCR4 by its diminishing effects on the desensitizing parameters of this axis. Although we found that Gi-activation through CXCR4 after a five- or 30-min ligand incubation time is unaffected by RAMPs, monitoring Gi activation kinetics with more sensitive methods might confirm these speculations.

Interaction between RAMPs and the class B receptors CLR and CTR involves the transmembrane domain as well as the folded extracellular domain of the receptor. Chemokine receptors lack a folded extracellular domain, and we show that ACKR3’s N-terminus is not required for RAMP regulation. In addition, activation by all ligands, including the small molecule VUF11207, predicted to bind within the orthosteric pocket of the receptor, is affected by RAMP3. This suggests that RAMP regulation involves transmembrane domain interactions with the receptor. More experiments are needed to pinpoint the exact mechanism by which RAMP3 affects receptor function, possibly by interfering with receptor dimer formation, dis-/allowing access to specific adaptor proteins, and/or stabilizing certain receptor conformations. Purification of ACKR3:RAMP complexes would allow the study of the RAMP-interaction and receptor conformation in the presence of RAMPs in more detail, *e.g.* by small molecule FRET, NMR, or cryo-EM.

As described previously, monitoring GPCR surface expression in the presence and absence of RAMPs has been a simple tool to screen for and expand the breadth of GPCR:RAMP interactions. Here, we showed that ACKR3 and CXCR4 surface expression is not affected by the presence of RAMPs, underlining that RAMP:GPCR interactions are versatile in their ability to chaperone GPCRs to the cell surface. This highlights the importance of more sophisticated methods for screening GPCR:RAMP interactions, including proximity-based assays to probe protein-protein interactions as performed by Lorenzen *et al.*, Mackie *et al*., and Kotliar *et al.* (17, 50, 51), but functional characterizations will be indispensable in characterizing the manifold RAMP: GPCR interactions and the receptor-specific effects of these interactions.

Finally, it should be noted that all experiments in this study have been performed in transiently transfected HEK293A cells. Interestingly, ACKR3 and RAMP3 (and RAMP2) expression patterns show significant overlaps in multiple cell types, including endothelial cells, smooth muscle cells, and fibroblasts. In contrast, RAMP1 is primarily expressed in endocrine cells, myocytes, and stromal cells, with little to no overlap with ACKR3 (or CXCR4) expression (52). This suggests that RAMP2 and RAMP3 could modulate ACKR3 function *in vivo* and might further partly explain why RAMP1 appears to be less relevant for either receptor’s functional regulation. As protein function can significantly differ between different cell types, more studies are needed to examine the exact effects of the ACKR3:RAMP interaction in these cells.

The pharmacological targeting of chemokine receptors has historically proven challenging (53). In the case of ACKR3, the finding that RAMPs can diminish receptor internalization and ligand scavenging presents a valuable opportunity to stabilize this interaction as a novel therapeutic strategy for pain management (5, 54) and against cancer (55, 56). Further dissecting the exact interactions and mechanisms by which RAMPs regulate the function of CXCR4, ACKR3, and other chemokine receptors might, therefore, facilitate the exploitation of this highly relevant protein class for therapeutic purposes.

## Experimental procedures

### DNA constructs

#### Receptor constructs

For mammalian cell expression in HEK293A cells, human ACKR3 and CXCR4 were cloned into a pcDNA vector containing the Rluc3 gene (Renilla luciferase A55T, C124A, M185V) at the C terminus of the receptor. Receptor-Rluc3 and GFP-10-β-arrestin2 were a kind gift from Nikolaus Heveker (Universite de Montreal, Montreal, QC, Canada).

The generation of the N-terminal cleavage mutant ACKR3Δ2-29, where residues 2 to 29 of ACKR3(-Rluc3) were removed, was described previously (44).

In SNAP-ACKR3 and SNAP-CXCR4, the receptor sequence was preceded by an N-terminal *IL2Rα-SS* signal sequence (*IL2Rα-SS: MDSYLLMWGLLTFIMVPGCQ*), followed by FLAG (DYKDDDDK) the SNAP-coding sequence.

For the SNAP-CLR construct, (SNAP-)ACKR3 was replaced by human CLR using the ClonExpress II One Step Cloning Kit (Vazyme, cat#C112). The sequence of all SNAP-constructs was *SS(IL2Rα) – FLAG – SNAP – receptor* (SS(*IL2Rα*): *MDSYLLMWGLLTFIMVPGCQ*, for membrane localization)

#### RAMP constructs

For the generation of 3xHA-RAMPs in the mammalian expression vector pcDNA3.1, pcDNA3.1 was linearized with HindIII-HF (New England Biolabs, cat#R3104) and XhoI (New England Biolabs, cat#R0146) restriction enzyme digest (NEB) and 3xHA-RAMP1-3 transferred to linearized pcDNA3.1 using the ClonExpress II One Step Cloning Kit (Vazyme, cat# C112). The final sequence of the constructs is SS-3xHA-RAMP, where SS is each RAMP’s endogenous signal sequence (1–27 for RAMP1 [NM_005855.4], 1 to 42 for RAMP2 [NM_005854.3] and 1 to 27 for RAMP3 [NM_005856.3]).

For the generation of the SNAP-RAMP constructs, (SNAP-)ACKR3 was replaced by RAMP1, RAMP2 or RAMP3 (each with removed endogenous signal sequence, see above) by Genscript cloning services.

All constructs were confirmed by sequencing.

Dynamin K44A was expressed in a pRK5 vector. The CAMYEL biosensor, mem-citrine-SH3, and the Rluc8-β-arrestin2-Sp1 were kindly provided by Jonathan Javitch (Columbia University).

### Cell culture

HEK293A (ThermoFisher, cat#R70507) cells were maintained at 5% CO_2_ and 37 °C in Dulbecco’s Modified Eagle Medium (DMEM) - GlutaMax (ThermoFisher, cat#31966) supplemented with 10% FBS and Pen-Strep mix (Merck, cat#P4333). For cell passaging, cells were washed in PBS and detached with 0.05% Trypsin-EDTA (VWR, cat#392-O459P).

### CXCL12 purification

CXCL12 was expressed and purified as previously described (57). In brief, CXCL12 preceded by N-terminal 8xHis-tag and an enterokinase cleavage site in a pET-21-based vector was expressed in *Escherichia coli*(DE3)pLysS (Agilent) and purified by affinity chromatography using Ni-NTA beads (Qiagen). The His-tag was removed by enterokinase (Bionordika) and CXCL12 was further purified with Ni-NTA beads and Grace Vydac C18 reverse-phase HPLC column (Avantor), followed by buffer exchange (20mM acetate pH 4), spin concentration and storage at −80 °C.

### Real-time internalization assay

The internalization assay was performed similarly as described previously (24). Briefly, DNA (final 40ng per well) was diluted in OptiMEM (ThermoFisher, cat#51985042), Lipofectamine2000 (ThermoFisher, cat#11668019) was added (final 0,075ng/well), and incubated for 10 min. The transfection mix was mixed with cells diluted in media (DMEM + 10% FBS + Pen-Strep) and 20.000 cells seeded in a white 384-well plate (*In vitro*, cat#GR-781201). For receptor internalization, 10ng SNAP-receptor DNA was filled up with 30ng pcDNA3.1 or 30ng RAMP DNA to a total of 40ng transfected DNA per well. For RAMP-internalization, cells were transfected with 20ng RAMP DNA and 20ng receptor-Rluc added depending on the setup. After 24 h, media was removed from the wells, and surface SNAP-protein labeled with 0.1 fmol/L SNAP-Lumi4-Tb (PerkinElmer, cat#SSNPTBD) in Opti-MEM for 60 min at 4 °C. Excess labeling reagent was removed by washing 6 times with internalization buffer (Hanks Balanced Salt Solution + 20mM HEPES + 1mM CaCl2 + 1mM MgCl2 + 1mg/ml BSA, pH 7.5). For quantification of cell-surface expression, an internalization buffer was added to wells and donor signals measured. For internalization-recording wells, Fluorescein-O′-acetic acid (Merck, cat#88596) was added to a final concentration of 25μM, followed by 10μl buffer or ligand and plate measurement @ 37 °C started immediately on a PerkinElmer EnVision Plate Reader.

Internalization ratios were calculated as follows:

$$Ratio\left(Internalization\right)=\frac{Emission\left(Donor,620nm,Lumi4-Tb\right)}{Emission\left(Acceptor,520nm,Fluorescein\right)}$$

Internalization was quantified by area under the curve (AUC) analysis in Graphpad prism (v10.2.3). SNAP-protein surface expression was quantified as the donor emission in absence of Fluorescein.

The final concentrations when recording internalization were 100nM CXCL12, 200nM CXCL11 (Peprotech, cat#300–46), 1μM VUF11207 (Biotechne/Tocris, cat#4780), 10μM TC14012 (Biotechne/Tocris cat#4300), 10μM adrenomedullin (1, 2, 3, 4, 5, 6, 7, 8, 9, 10, 11, 12, 13, 14, 15, 16, 17, 18, 19, 20, 21, 22, 23, 24, 25, 26, 27, 28, 29, 30, 31, 32, 33, 34, 35, 36, 37, 38, 39, 40, 41, 42, 43, 44, 45, 46, 47, 48, 49, 50, 51, 52) (Bachem, cat#H-2932), 10μM PAMP-12 (Biotechne/Tocris, cat#6551).

#### β-arrestin recruitment assay

For the direct β-arrestin recruitment assay, 500.000 cells per well were seeded in a 6-well plate and allowed to adhere for 24 h. Cells were transfected in FBS-free DMEM-GlutaMax with 50ng of receptor-Rluc3 DNA and 500ng GFP-β-Arrestin2. 150ng of RAMP1-, RAMP2- or RAMP3-encoding DNA was added (1:3 receptor:RAMP DNA ratio) and pcDNA3.1 was added to a total transfected DNA amount of 1μg/well. Transfection was performed by polyethyleneimine (PEI) (Polysciences, cat#23966) in a ratio of 1:2 (μg DNA:μg PEI). 24h after transfection, media was exchanged with DMEM +10% FBS + Pen-Strep, and cells recovered for another 24h.

On day 4, cells were washed with PBS and mechanically lifted in BRET buffer (PBS + 0,1% Glucose). 100.000 cells were seeded in a white 96-well plate, incubated for 45 min at 37 °C and appropriately diluted ligand (in BRET buffer) was added to each well. 30 min after incubation with ligand, Coelenterazine 400a (DeepBlueC) (VWR/Cayman, cat#CAYM16157) was added to a final concentration of 5μM, and the plate read on a PerkinElmer EnVision Plate Reader. BRET values were calculated as follows:

$$Ratio\left(BRET\right)=\frac{Emission\;\left(Acceptor,535nm,GFP\right)}{Emission\left(Donor,400nm,Rluc\right)}$$

For quantification of total receptor expression, mean donor values were analyzed as follows:

Receptorexpression=Emission(400nm,donor,Rluc)

For the indirect (bystander) β-arrestin recruitment assay, cells (0.35M/ml) were directly combined with transfection mix containing 200ng ST-receptor, 50ng Rluc8 – β-arrestin2 – Sp1 and 800ng mem-citrine-SH3 (58), 100μl/well of the cell/transfection mix plated in a coated 96-well plate. After 48 h of transfection, cells were washed with PBS, substrate (Coelenterazine H, Nanolight technology, cat#301–10) and ligand added and plate read after 30 min.

All dose-response curves were fitted with a 4-parameter nonlinear regression model (variable slope) in Graphpad prism (v10.2.3) and efficacy and baseline values were extracted from top and bottom fit, respectively. Potencies were extracted from LogEC50 values of the fits.

### CXCL12 scavenging assay (ELISA)

To determine the CXCL12 scavenging, HEK293A cells were seeded and transfected similarly as described for the β-arrestin recruitment assay. Specifically, cells were transfected with 200ng ACKR3-Rluc3 and 600ng RAMP1,2 or 3. On day 4, cells were resuspended in regular cell media and 100.000 cells plated in a 96 well plate (in duplicates). After 6 h of readhering, CXCL12 was added to a final concentration of 25nM and incubated overnight (37 °C). The next day, the CXCL12 concentration in the cell supernatant was quantified using an ELISA kit (Biotechne/RnD systems, cat#DSAOO) according to the manufacturer’s instructions.

Receptor expression was quantified by addition of 5μM Coelenterazine 400a (VWR/Cayman, cat#CAYM16157) to the cells and subsequent plate measurement of emission at 400nm (luciferase emission).

Scavenged CXCL12 by ACKR3 in absence and presence of RAMPs was corrected for differences in receptor expression according to following calculation:

$$CXCL12_{scavenged}=\left(CXCL12_{remaining,pcDNA}-CXCL12_{remaining,sample}\right)*\frac{expression_{receptor\;alone}}{expression_{sample}}$$

### cAMP accumulation assay

To quantify the inhibition of cAMP by CXCR4, HEK293A cells were seeded and transfected similarly as described for the β-arrestin recruitment assay. Specifically, cells were transfected with 200ng CXCR4 (−/+ RAMPs, 1:3 ratio CXCR4: RAMP DNA) and 500ng of the BRET-based cAMP sensor CAMYEL (59).

On assay day, the substrate Coelenterazine H (Nanolight technology, cat#301–10) was added to a final concentration of 5μM and cAMP production was stimulated by treatment with 1.5μM forskolin (Merck, cat#F6886). CXCL12 was added at different concentrations for five or 30 min before plate reading on a PerkinElmer EnVision Plate Reader.

cAMP inhibition was measured as follows:

$$Ratio\left(BRET\right)=\frac{Emission\left(Acceptor,530nm\right)}{Emission\left(Donor,480nm\right)}$$

For time-resolved measurements of cAMP accumulation, cells were incubated with Forskolin for 30 min, substrate and ligand added, and plate read immediately.

### Flow cytometry

Cells were seeded and transfected with 50ng ACKR3-Rluc3 (−/+ 150ng RAMP) in the same way as described for the β-Arrestin assay. On day 4, cells were washed (PBS +0.5% BSA), counted, and plated in a 96-well plate (round bottom wells). Cells were stained with 0.75μl/well of Human/Mouse CXCR7/RDC-1 PE-conjugated Antibody (R&D systems, cat# FAB4227P) and 0.1μg/well of FITC-conjugated Anti-HA antibody (1h @4 °C), washed three times (PBS + 0.5% BSA) and fixed (PBS + 3.7% PFA) and samples measured on a LSRFortessa (3 laser, BD Life Sciences) with compensation performed with FACSDiva software (BD Life Sciences). Results were analyzed using FlowJo Software (BD Life Sciences, v10.8).

### Western blot from lysed, transfected cells

Cells were seeded and transfected as described for the β-Arrestin recruitment assay (or with 175.000 cells seeded per well in a 12-well plate), with 150-200ng (FLAG-)SNAP-receptor and 450-600ng HA-RAMP or pcDNA3.1 added (3:1 receptor: RAMP ratio). On day 4, media was aspirated, cells washed in 1ml PBS and resuspended in 100μl 1x RIPA buffer (Merck/Sigma, cat#20–188) (+cOmplete mini protease inhibitor cocktail (Merck/Sigma, cat#11836153001)). Lysis was incubated for 30 min and protein concentration in the supernatant was quantified by Pierce Rapid Gold BCA assay (ThermoFisher, cat#A53225) according to the manufacturer’s protocol. 40μg protein was loaded on a Criterion TGX Precast 4 to 15% SDS gel (Bio-Rad, cat#5671084) and subsequently transferred to an LVDF membrane (Bio-Rad, cat#1704275), followed by staining with Monoclonal ANTI-FLAG M1 antibody produced in mouse (for detection of ST-receptors or ST-RAMPs) (Merck, cat#F3040) or monoclonal Anti-HA mouse antibody (for detection of HA-RAMPs) (Merck, cat#H3663) and secondary IRDye 800CW Goat anti-mouse IgG secondary antibody (Li-COR, cat#925–32210). Membrane was imaged on a Licor Odyssey. ImageJ was used to quantify expression from the blot images.

### Data analysis

Dose-response curves were fitted with a four-parameter nonlinear regression model (log(agonist) vs. response, with variable slope). To account for differences between experiment days, the means of multiple experiments were compared by ordinary two-way ANOVA of main column effects with Dunnett’s correction for multiple testing. All data were analyzed with Graphpad Prism software (v10.2).

## Data availability

All data are available upon request to martin@sund.ku.dk.

## Supporting information

This article contains supporting information.

## Conflict of interest

The authors declare the following financial interests/personal relationships which may be considered as potential competing interests: M.M.R. is co-founder of Synklino ApS. All other authors declare no conflict of interest.
