# Supplementary material for: Regulation of the chemokine receptors CXCR4 and ACKR3 by receptor activity-modifying proteins
Source: J Biol Chem. 2024 Dec 9;301(1):108055. doi: 10.1016/j.jbc.2024.108055 (PMC11760809; doi:10.1016/j.jbc.2024.108055)
Supplement: Supporting information [file mmc1.pdf]

Regulation of the chemokine receptors CXCR4 and ACKR3 by receptor activity-modifying proteins

Fabian Pfersdorf[1], Lucas Romanazzi[1], Mette Marie Rosenkilde[1], Martin Gustavsson[1*]

[1]Department of Biomedical Sciences, University of Copenhagen, Copenhagen, Denmark
* Corresponding author. Correspondence to: martin@sund.ku.dk

- Supporting information -

Running title:
RAMP regulation of CXCR4 and ACKR3

Keywords:
G protein-coupled receptor (GPCR), chemokine receptor, receptor activity-modifying proteins (RAMPs), ACKR3, CXCR4, CXCL12, protein-protein interaction, receptor internalization, arrestin recruitment, signal transduction

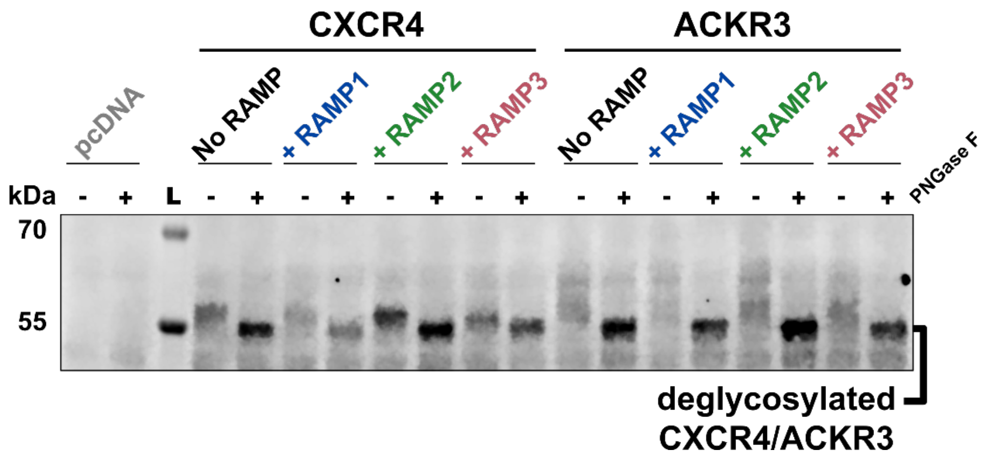

Figure S1: Representative western blot for quantification of total expression. ST-CXCR4 and ST-ACKR3 were expressed alone or together with different RAMP variants and samples treated with PNGaseF for removal of N-linked glycosylations.

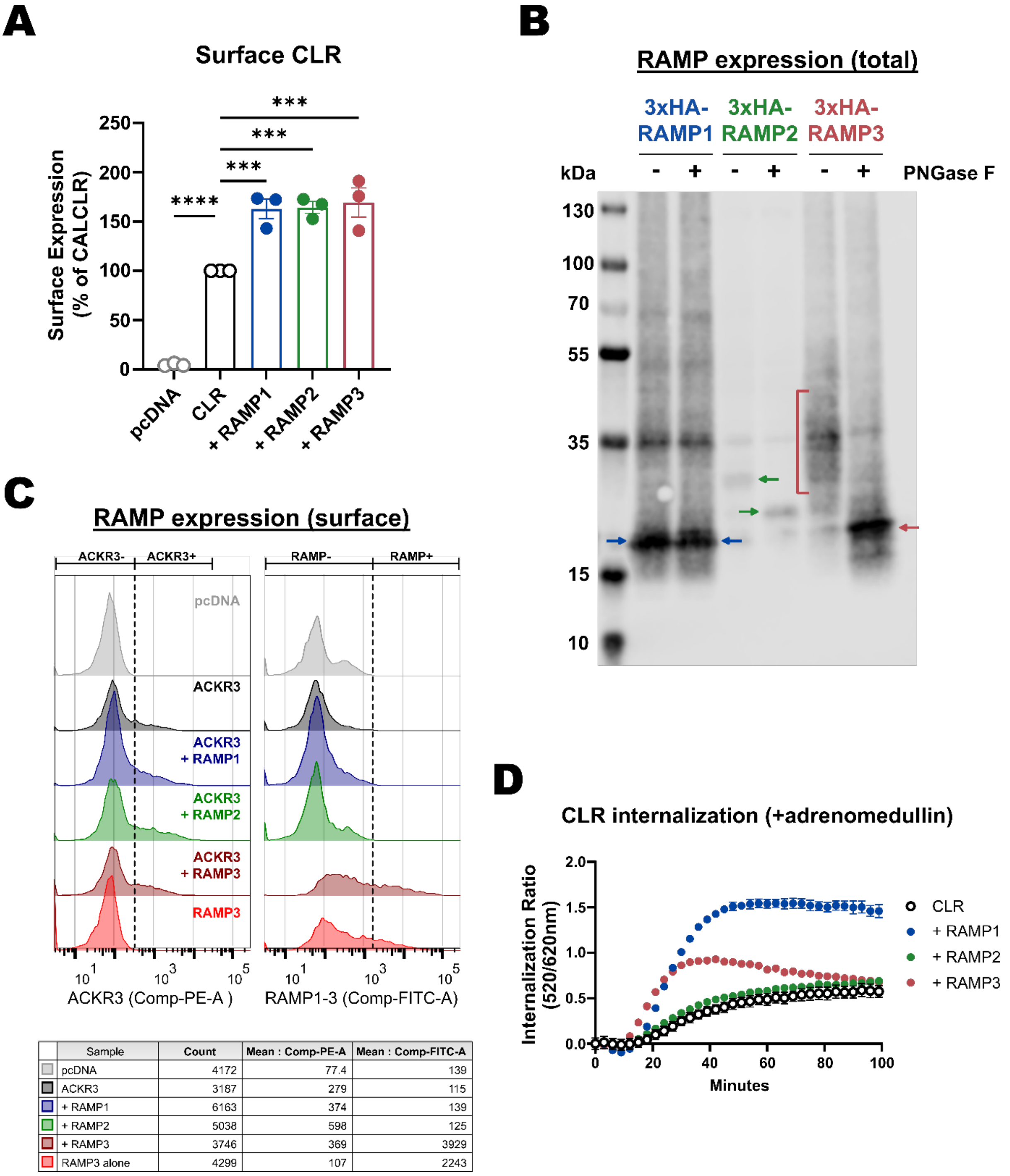

Sample	Count	Mean : Comp-PE-A	Mean : Comp-FITC-A
pcDNA	4172	77.4	139
ACKR3	3187	279	115
+ RAMP1	6163	374	139
+ RAMP2	5038	598	125
+ RAMP3	3746	369	3929
RAMP3 alone	4299	107	2243

Figure S2: CLR internalization & HA-RAMP expression. (A) SNAP-surface labeling of CLR -/+ RAMP1-3, shown as mean ± SEM percentage of CLR alone (n=3). **(B)** Representative western blot of HA-RAMP1-3 from transfected HEK293 cells, both without and after treatment with PNGase F to remove N-linked glycosylation. Arrows indicate HA-RAMP bands (theoretical MWs: RAMP1 ~17.3kDa, RAMP2 ~18.6kDa, RAMP3 ~17.2kDa). **(C)** Histograms showing flow cytometry surface staining of transfected HEK293A cells. **(D)** CLR-internalization -/+ RAMP1-3 in response to adrenomedullin (1µM). Data points shown are mean ± SEM of independent experiments (n=3). Statistical analysis was performed on the non-normalized data by ordinary two-way ANOVA of main effects with Dunnett's correction for multiple testing. (****$p < 0.0001$, ***$p < 0.001$).

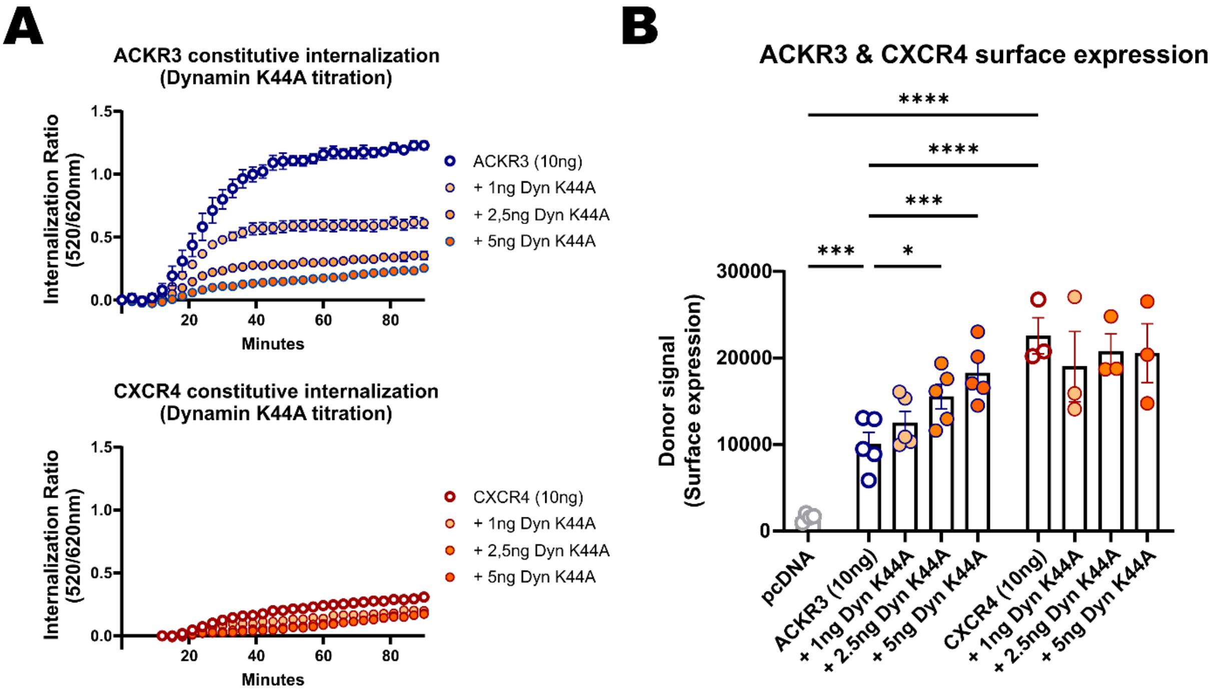

Figure S3: CXCR4 and ACKR3 surface expression and constitutive internalization + Dynamin K44A. (A) SNAP-surface labeling to quantify surface expression of CXCR4 and ACKR3, co-transfected with increasing amounts of Dyn K44A. Bars show mean ± SEM of independent experiments (n=3-5), with each experiment shown as a single datapoint. Statistical analysis was performed by ordinary two-way ANOVA of main effects with Dunnett's correction for multiple testing. (****p < 0.0001, ***p < 0.001, **p < 0.01, *p < 0.05, - = ns). **(B)** Constitutive internalization of CXCR4 and ACKR3 when co-transfected with increasing amounts of Dyn K44A (mean ± SEM of independent experiments, n=3-5).

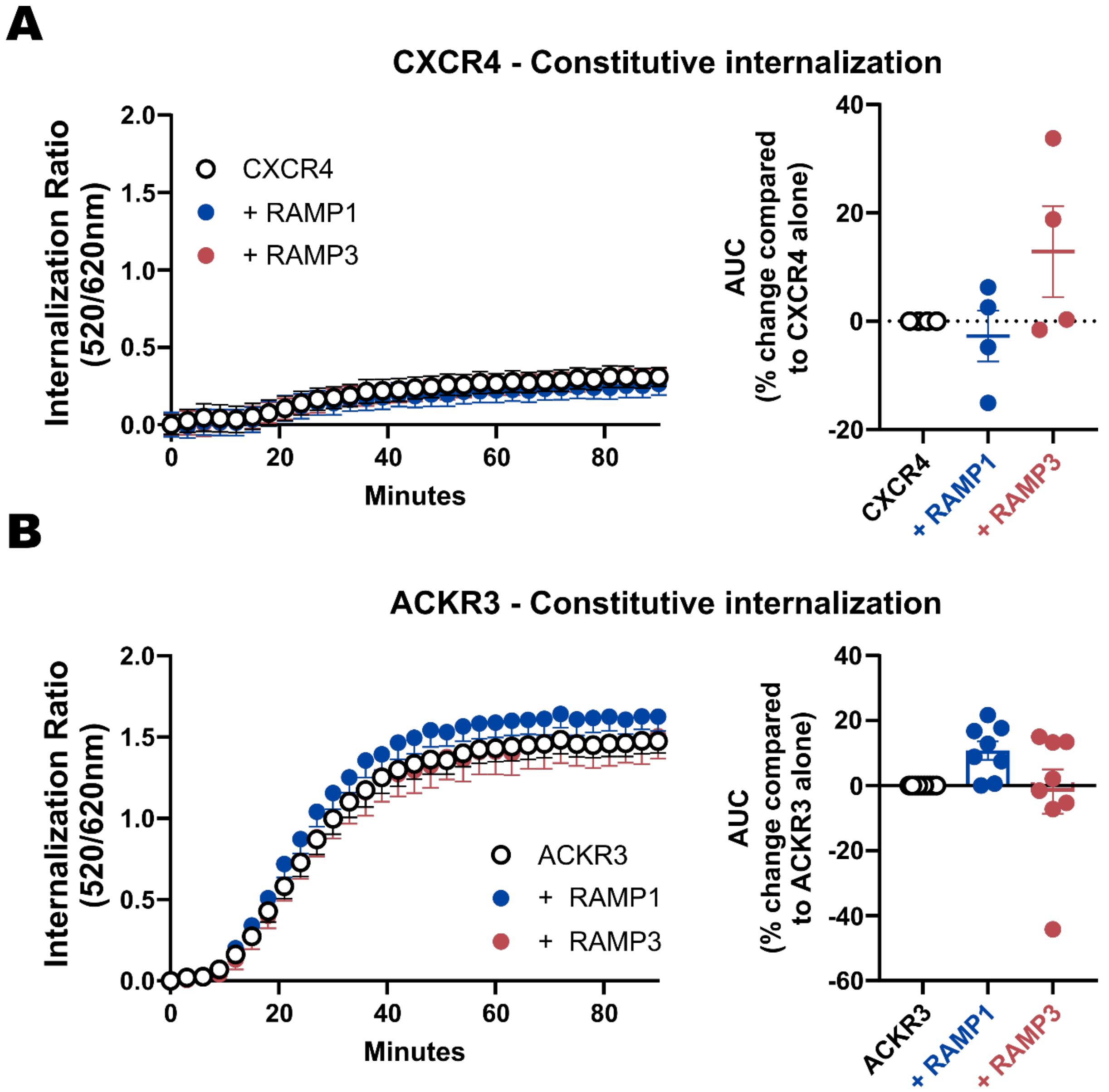

Figure S4: CXCR4 and ACKR3 constitutive internalization -/+ RAMPs. Internalization curves are represented as mean ± SEM of independent experiments (n=4 for CXCR4, n=8 for ACKR3), and AUC (90 minutes) of each experiment plotted as a single datapoint in bar graphs (right). Bars represent mean ± SEM of independent experiments. Statistical analysis was performed by ordinary two-way ANOVA of main effects with Dunnett's correction for multiple testing. (****p < 0.0001, ***p < 0.001, **p < 0.01, *p < 0.05, - = ns).

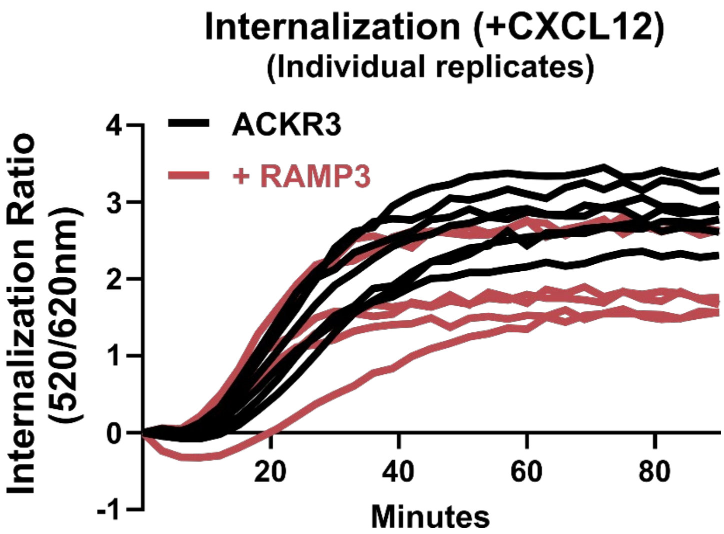

Figure S5: CXCL12-induced internalization of ACKR3 (-/+ RAMP3). Individual experiments (n= 6-7, each performed in technical triplicates) are shown as black (ACKR3) and red (+RAMP3) lines.

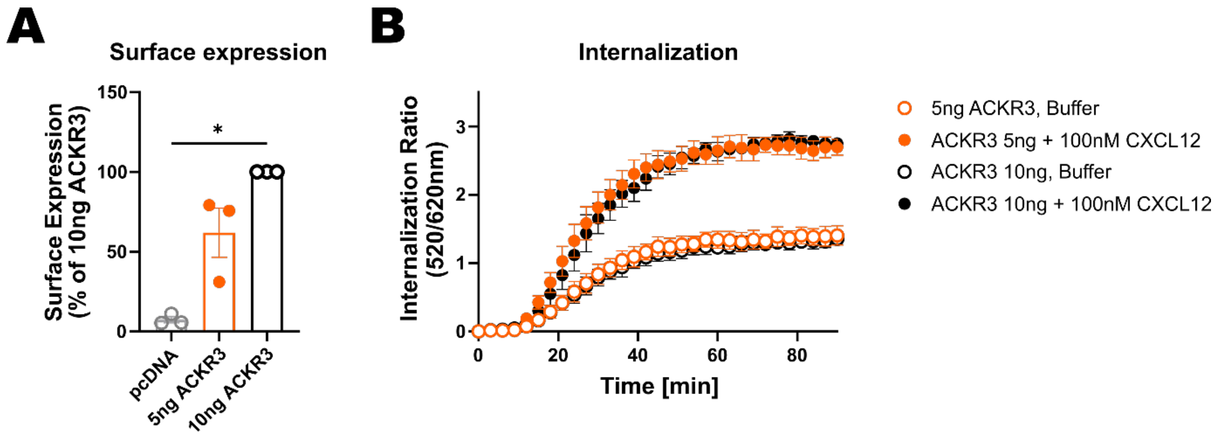

Figure S6: ACKR3 titration & internalization. (A) ACKR3 surface expression of cells transfected with 0ng (gray), 5ng (orange) or 10ng (black) of SNAP-ACKR3, with bars indicating mean ± SEM of independent experiments (n=3). **(B)** Internalization curves of 5ng and 10ng transfected DNA amount encoding SNAP-ACKR3 in absence and presence of 100nM CXCL12 are represented as mean ± SEM of independent experiments (n=3). Statistical analysis was performed by ordinary two-way ANOVA of main effects with Dunnett's correction for multiple testing. (*$p < 0.05$, - = ns).

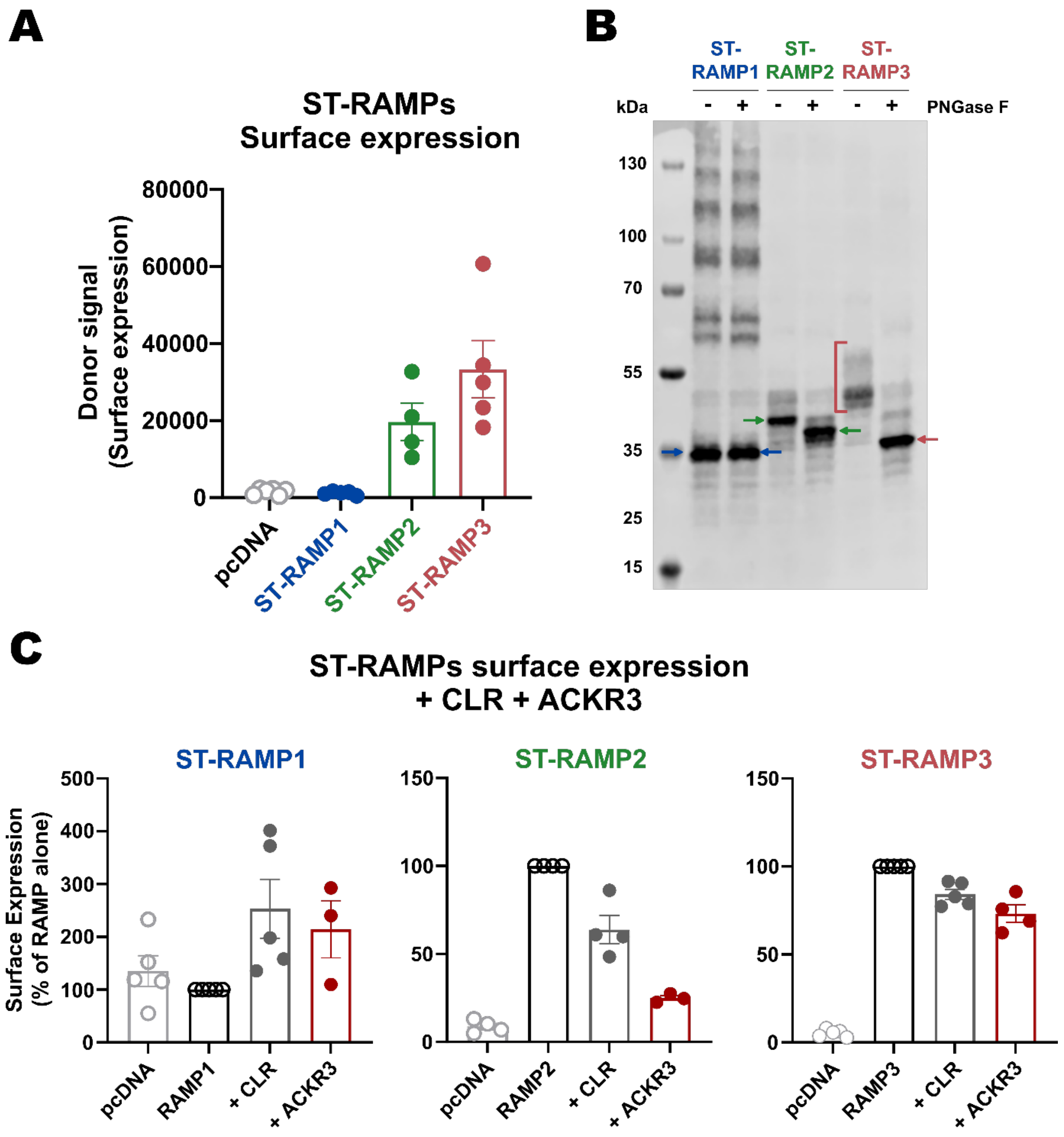

Figure S7: Expression of SNAP-RAMP constructs. (A) Surface expression of SNAP-RAMP1-3 determined by SNAP-surface labelling. **(B)** Western blot of solubilized cells transfected with SNAP-RAMP1-3, indicating total expression. Samples were treated with PNGase F to remove N-linked glycosylations. Theoretical MWs: SNAP-RAMP1 ≈ 34.8 kDa, SNAP-RAMP2 ≈ 36.1 kDa, SNAP-RAMP3 ≈ 34.7 kDa. **(C)** SNAP-surface labelling of SNAP-RAMP1-3 in absence (black, open circles) and presence of CLR (gray) or ACKR3 (red). Data are presented as percentage of SNAP-RAMP alone, bars indicate mean ± SEM of independent experiments (n=3-5).

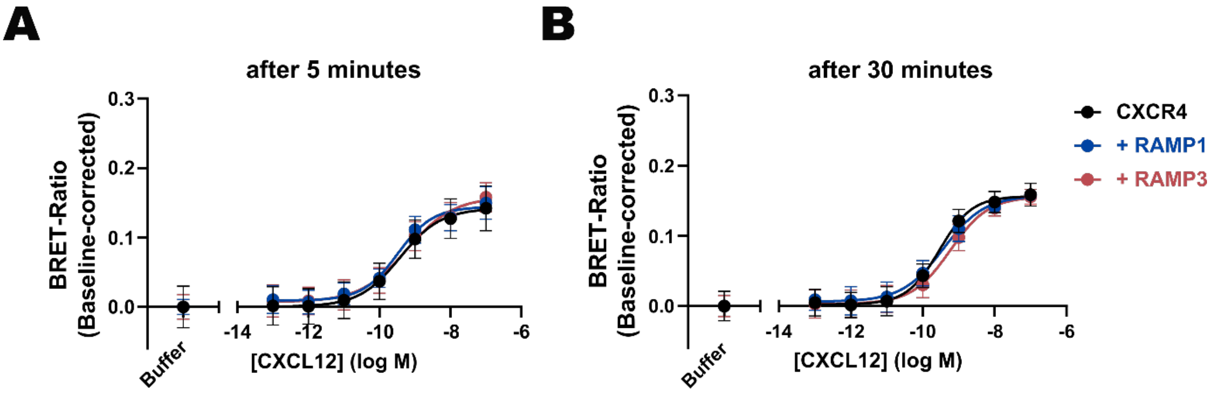

Figure S8: Inhibition of cAMP accumulation by CXCR4 (-/+ RAMPs). CXCR4 was co-expressed with the cAMP sensor (CAMYEL) and RAMPs, cells treated with forskolin to increase cellular cAMP levels, and cAMP inhibition through CXCR4 stimulated by CXCL12 for 5 **(A)** or 30 minutes **(B)**. Data are baseline-corrected for buffer condition and presented as mean ± SEM of independent experiments (n=3).

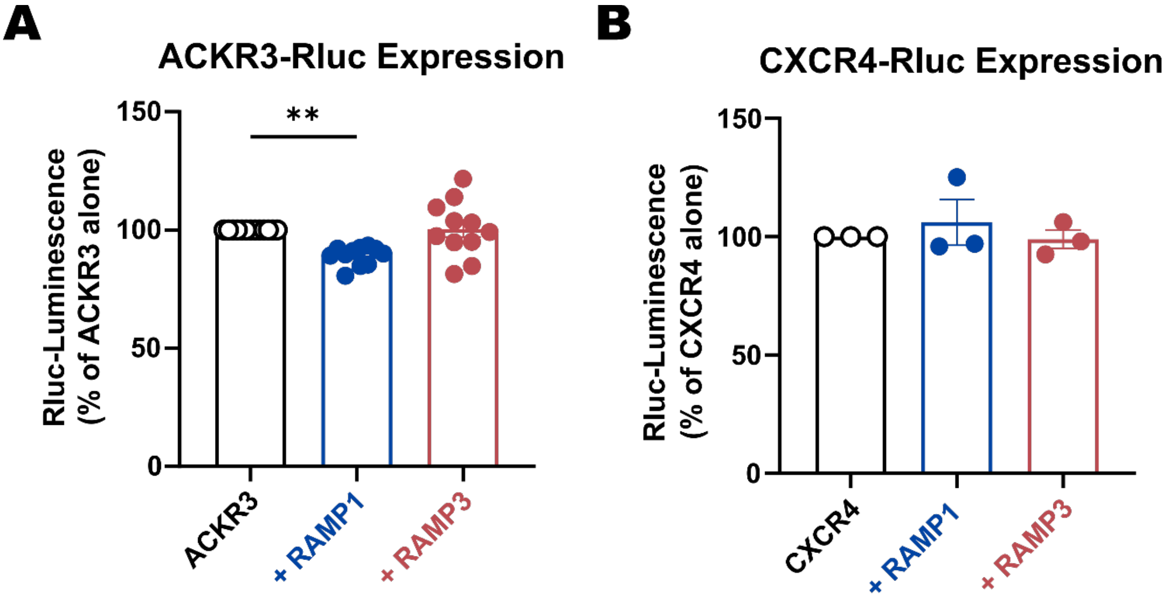

Figure S9: Total expression of receptor-Rluc constructs -/+ RAMP1&3. Data are normalized to receptor alone and bars represent mean ± SEM of independent experiments (n=11 for ACKR3 (A), n=3 for CXCR4 (B)). Statistical analysis was performed on the non-normalized data by ordinary two-way ANOVA of main effects with Dunnett's correction for multiple testing. (**p < 0.01, - = ns)

.

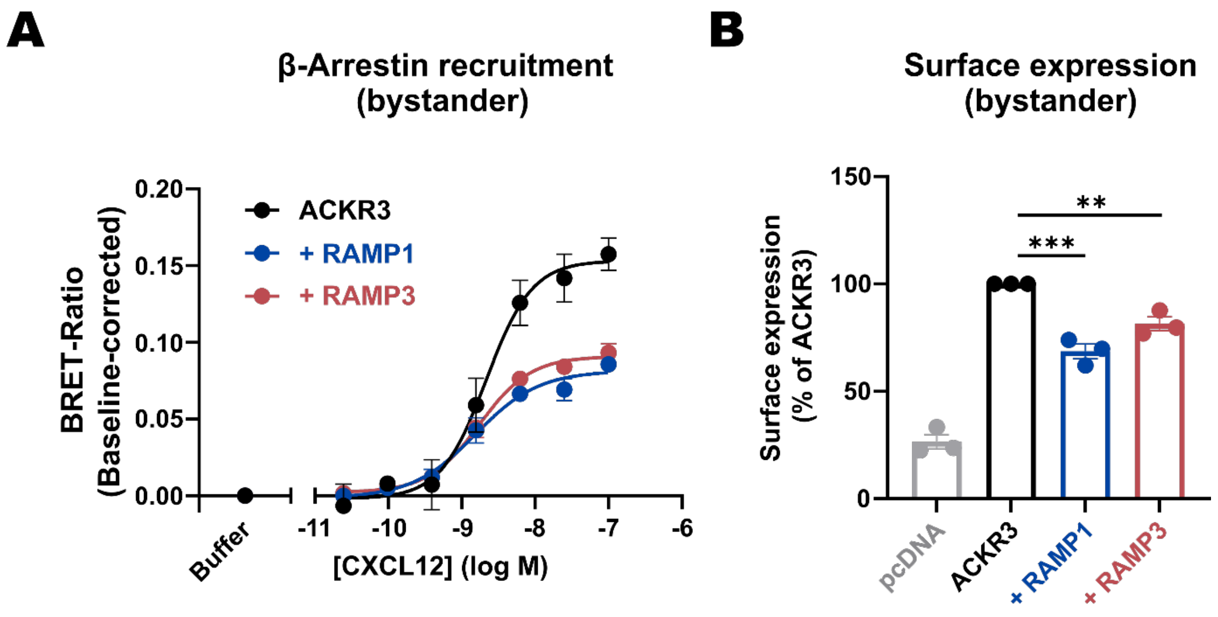

Figure S10: β-arrestin2 recruitment (bystander BRET assay). (A) Recruitment of β-arrestin2-Rluc to mem-citrine when ACKR3 was expressed alone or with RAMP1/3 and stimulated with CXCL12. (B) Surface expression was quantified by SNAP-surface labeling. Statistical analysis was performed by ordinary two-way ANOVA of main effects with Dunnett's correction for multiple testing. (***$p < 0.001$, **$p < 0.01$)

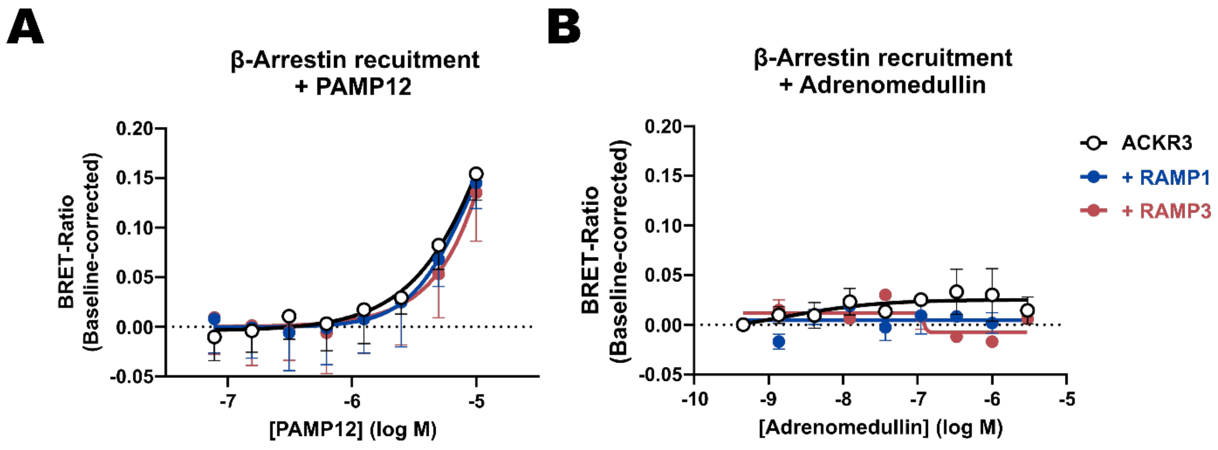

Figure S11: β-arrestin recruitment to ACKR3 induced by PAMP-12 (A) and Adrenomedullin (B). Data are baseline-corrected and presented as mean ± SEM (A, n=3)) or as a representative experiment showing mean + SD (B, n=1).
